# Impact of a modified Broviac maintenance care bundle on bloodstream infections in paediatric cancer patients

**DOI:** 10.3205/dgkh000258

**Published:** 2015-11-16

**Authors:** Rhoikos Furtwängler, Carolin Laux, Norbert Graf, Arne Simon

**Affiliations:** 1 Department of Paediatric Oncology and Haematology, University Hospital, Homburg/Saar, Germany

**Keywords:** bacteraemia, Broviac, children with cancer, preventive protocol

## Abstract

**Background:** During intensive chemotherapy, bloodstream infection (BSI) represents an important complication in paediatric cancer patients. Most patients carry a long-term central venous access device (CVAD). Improved maintenance care of these vascular catheters may decrease the risk of BSI.

**Methods:** Intervention study (adapted CVAD prevention protocol) with two observation periods (P1: 09-2009 until 05-2011; P2: 09-2011 until 05-2013); prospective surveillance of all laboratory confirmed BSIs. In P2, ready to use sterile NaCl 0.9% syringes were used for CVAD flushing and octenidine/isopropanol for the disinfection of catheter hubs and 3-way stopcocks.

**Results:** During P1, 84 patients were included versus 81 patients during P2. There were no significant differences between the two patient populations in terms of median age, gender, underlying malignancy or disease status (first illness or relapse). Nearly all CVADs were Broviac catheters. The median duration from implantation to removal of the CVAD was 192 days (Inter-quartile-range (IQR); 110–288 days) in P1 and 191 days (IQR; 103–270 days) in P2. 28 BSI were diagnosed in 22 patients in P1 (26% of all patients experienced at least one BSI) and 15 BSI in 12 patients in P2 (15% of all patients). The corresponding results for incidence density (ID) were 0.44 (CI95 0.29–0.62) for P1 vs. 0.34 (0.19–0.53) BSI per 100 inpatient days for P2 and for incidence rate (IR) 7.76 (5.16–10.86) in P1 vs. 4.75 (2.66–7.43) BSI per 1,000 inpatient CVAD utilization days. In P1, 9 BSI were caused by CoNS vs. only 2 in P2 (IR 2.49; CI95 0.17–4.17 vs. 0.63; CI95 0.08–1.72). In P1 two BSI (7%) lead to early removal of the device. During P2 one CVAD was prematurely removed due to a Broviac-related BSI (6.7%).

**Conclusion:** The preventive protocol investigated in this study led to a reduction of BSI in paediatric cancer patients. This result was clinically relevant but – due to insufficient power in a single centre observation – the difference did not reach statistical significance. The most pronounced trend in BSI reduction was observed for CoNS infections. Thus, improving maintenance care of the CVAD may result in lower CVAD-linked infection rates. The higher acquisition cost of the ready to use NaCl 0.9% flushing syringes and octenidine/propanol hub disinfection were probably balanced by cost savings in the intervention period.

## Zusammenfassung

**Hintergrund:** Während der intensiven Chemotherapie sind bei Kindern mit Krebserkrankungen Blutstrominfektionen (BSI) eine potentiell lebensbedrohliche Komplikation. Die meisten Patienten haben einen dauerhaft implantierten oder getunnelten zentralen Venenkatheter (CVAD, z.B. Broviac). Ein verbessertes Protokoll der Erhaltungspflege kann möglicherweise die Rate von BSI senken.

**Methode:** Interventionsstudie zum Nutzen eines modifizierten Präventionsbündels in zwei prospektiven Beobachtungszeiträumen (P1: bis 05-2011; P2: 09-2011 bis 05-2013); prospektive Surveillance aller mikrobiologisch gesicherten BSI. In P2 wurden fertig konfektionierte NaCl 0.9% Spritzen (10 ml) zum Spülen des Broviacs verwendet. Außerdem wurden alle Konnektions- und Zuspritzstellen (Dreiwegehähne) mit Octenidin/Propanol desinfiziert.

**Ergebnisse:** Während P1 wurden 84 Patienten und während P2 81 eingeschlossen. Die beiden Patientenpopulationen zeigten keine signifikanten Unterschiede in Bezug auf das Alter, die Grunderkrankung oder ihren Status (Anteil mit Rezidiv). Praktisch alle CVAD waren Broviac-Katheter. Die mediane Nutzungsdauer lag in P1 bei 192 d (Interquartile-Range (IQR); 110–288 Tage) und in P2 bei 191 d (IQR; 103–270 Tage). In P1 wurden insgesamt 28 BSI bei 22 Patienten diagnostiziert (Inzidenz für mind. 1 BSI 26%), in P2 waren es nur 15 BSI bei 12 Patienten in P2 (Inzidenz 15%). Die entsprechenden Ergebnisse für die Inzidenzdichte lagen bei 0.44 (CI95 0.29–0.62) BSI pro 100 stationäre Patiententage für P1 vs. 0.34 (0.19–0.53) für P2. Die Inzidenzrate (IR) pro 1.000 stationäre Anwendungstage lag bei 7.76 (5.16–10.86) für P1 vs. 4.75 (2.66–7.43) für P2. Während in P1 9 BSI durch CoNS detektiert wurden, waren es in P2 nur 2 (IR 2.49; CI95 0.17–4.17 vs. 0.63; CI95 0.08–1.72).

**Fazit:** Das modifizierte Präventionsbündel führte zu einer Reduktion von BSI bei pädiatrisch onkologischen Patenten mit Broviac Katheter. Wie in einigen Studien U.S.-amerikanischer Zentren war auch hier die Fallzahl im Beobachtungszeitraum von insgesamt 42 Monaten zu limitiert für ein statistisch signifikantes Ergebnis. Trotzdem sind diese Ergebnisse klinisch von hoher Relevanz. Wahrscheinlich können insbesondere BSI durch CoNS verhindert werden.

## Introduction

In paediatric patients with cancer, alterations in host defence mechanisms against infection are related to the underlying illness (e.g. haematologic malignancy), to intensive treatment with immunosuppressive drugs (neutropenia, lymphocytopenia), and to additional side effects such as gastrointestinal mucositis or graft versus host disease (GVHD) [1], [2]. In this setting, fever with or without neutropenia is an important therapy-associated complication [3]. Besides fever without a detectable focus, which accounts for up to 60% of all cases, and a wide spectrum of clinically or microbiologically defined infections, bacteraemia due to Gram-positive and Gram-negative pathogens significantly affect morbidity and mortality in this high risk population [4]. Most patients with bacteraemia have a long-term central venous access device (CVAD) in use. ‘CVAD’ refers to tunnelled Broviac/Hickman or subcutaneously implanted port catheters [4], [5], [6], [7]. These devices are of proven benefit for patients and caregivers but their use increases the risk of bacteraemia [8], [9].

Patients may experience the onset of bacteraemia as in- or as outpatients, since paediatric cancer patients are discharged from hospital as often as possible even during intensive chemotherapy treatment periods [10], [11].

The prospective surveillance of CVAD-associated and CVAD-related bacteraemia with adapted case definitions and standardised methods for data analysis and reporting has been established at our paediatric cancer centre since September 2008 as quality management initiative [6], [12]. Surveillance efforts aim at the identification of critical control points for the reduction of health-care associated infections in paediatric cancer patients [5], [6], [13], [14], [15]. In the long term, prospective surveillance may be used to investigate the effect of preventive intervention protocols [16] or the impact of new medical products on infection rates [17].

Paediatric cancer treatment centres still differ substantially in approaching the management and care of CVADs [18], [19], [20]. Unfortunately, it remains an unresolved issue, which combination of preventive strategies is most effective in reducing CVAD-associated infection rates [18], [21], [22], [23].

Herein, data derived from prospective surveillance of bloodstream infections during two observation periods is reported from a German paediatric cancer centre. One aim of this report is to elucidate the impact of relatively small changes in CVAD maintenance care (‘preventive protocol’) on patients’ safety.

## Methods

About 40–50 paediatric cancer patients are admitted per year with newly diagnosed or relapsed malignancies to the Homburg/Saar university affiliated paediatric cancer treatment centre. The centre runs a 12 bed inpatient unit and a specialised outpatient clinic [20]. Anticancer treatment of childhood malignancies refers to the cooperative protocols of the German Society for Paediatric Oncology and Haematology (GPOH). In patients with acute leukaemia, the centre adheres to protocols derived from the international BFM group.

Fever was defined as body temperature >38.5^o^C for at least 4 hours or once >39^o^C. Neutropenia was defined as a total number of granulocytes <0.5 x 10^9^/L or a total number of leukocytes <1.0 x 10^9^/L without differential counts available. 

In this study, two central venous blood culture samples (aerobic and anaerobic) were collected from patients with fever under aseptic conditions and after disinfection of the CVAD hub before the first dose of intravenous antibiotics. In patients with bilumen Broviac catheter, two culture bottles were filled from each lumen.

Blood cultures were processed using the BD BACTEC™ automatic detection system (Beckton Dickinson, Heidelberg) and species differentiation according to standard microbiological procedures [24].

Bacteraemia (bloodstream infection; BSI) referred to the growth of a bacterial pathogen in blood culture derived from a patient with fever or other signs of infection. At least two positive blood culture bottles drawn from the CVAD were stipulated to accept coagulase-negative staphylococci (CoNS) as pathogens in this clinical context. ‘CVAD-associated BSI’ referred to a patient with BSI, a CVAD in use and no evidence of an alternative primary focus of infection. To allocate the BSI to the category ‘CVAD-related infection’ blood cultures taken from the device had to be repeatedly positive for longer than 72 hours or the bacteria were detected on the catheter tip after premature removal of the device. In case of patients with microbiologically or clinically defined primary focus of infection, the corresponding BSI was allocated as secondary bacteraemia. A BSI not related to the CVAD and probably caused by a distinct clinically defined focus of infection was termed ‘secondary BSIs’.

The prospective Oncoped tool for the surveillance of healthcare-related infections in paediatric cancer patients in Germany has been previously described in detail [5], [6], [12], [25]. Incidence densities (ID; BSI per 100 inpatient days) and incidence rates (IR; BSI per 1,000 CVAD utilization days) were calculated. Clinical severity of the BSI events was graded according to consensus criteria published by Goldstein et al. [26].

Table 1 (Tab. 1) shows basic issues of CVAD maintenance care and changes in certain practices to prevent bacteraemia implemented in the two different study periods. In Period 2, 10 ml sterile NaCl 0.9% ready to use syringes, (BD PosiFlush™) were used for flushing of the CVAD instead of manually prepared NaCl 0.9% syringes [27]. This decision was made to decrease the risk of contamination of the flushing solution [28]. In addition, the minimal frequency of i.v. system changes and of routine Broviac flushing were extended (from 48 to 96 hours and from twice to once a week, respectively). Both strategies aim to reduce any manipulation at the catheter hub [29]. A lower number of manipulations reduce the chance of manual contamination and subsequent bacterial colonisation of the CVAD lumen [30]. Furthermore, the antiseptic (Octeniderm™) was used in period 2 to disinfect the hub and any other injection site (e.g. 3-way stopcocks) before access or disconnection [31]. 

Comparable to 2% chlorhexidine in 70% isopropanol, Octeniderm™ combines a fast acting mixture of 0.1 g octenidine, a broad spectrum biguanide antiseptic with remanence effect, with 30.0 g 1-propanol and 45.0 g 2-propanol in 100 g solution [32], [33], [34], [35], [36]. Last but not least, the importance of hand hygiene (hand disinfection; HD) [37] was reemphasized in clinical rounds, educational sessions and a hospital wide campaign driven by infection control nurses.

The study protocol was approved by the ethics committee of the medical association of the Saarland/Germany (Ref-No. 158/11). Informed consent to participate in the collection and analysis of surveillance data was obtained according to our institutional policies from patients or their legal guardians.

Statistic analysis was carried out using Windows Excel 2007 and SPSS; 95% confidence intervals were calculated as discussed by Pearson and Clopper [Biometrika 1934, 26 (4) 404-13]. Mann-Whitney-, Chi-Square- and Fisher’s exact Test (in case of n<5 in at least a single cell) were calculated for non-parametric and binomial variables respectively on SPSS. The significance level (error probability level alpha) was 5%.

## Results

Two 21 months’ time periods were compared. All eligible patients participated in the study. During period 1, 84 patients were included, versus 81 patients during period 2. The basic patients’ characteristics are shown in Table 2 (Tab. 2). There were no significant differences between the two patient populations in terms of median age, gender, underlying malignancy or disease status (first illness or relapse).

In period 1, 9 patients (11%) had no CVAD. Nearly all patients with a CVAD (73 of 76; 96%) had a Broviac in use; only 3 (4%) of all patients had a port implanted. The cumulative number of in- and outpatient days with a CVAD (Broviac or port) in period 1 were 16,350 days. For Broviac CVADs, the median duration from implantation to removal was 192 days (IQR; 110–288 days).

In period 2, 11 patients (13.6%) had no CVAD. Nearly all patients with a CVAD in period 2 (68 of 70; 97%) had a Broviac in use; only 2 (2.5%) of all patients had a port implanted. The cumulative number of in- and outpatient days with a CVAD (Broviac or port) in period 2 were 14,304 days. For Broviac CVADs, the median duration from implantation to removal was 191 days (IQR; 103–270 days).

During the prospective surveillance study, 28 BSI were diagnosed in 22 patients in period 1 (26% of all patients experienced at least one BSI). The corresponding numbers in period 2 were 15 BSI in 12 patients (15% of all patients).

The IR (BSI CoNS) was reduced by 75% and the IR (BSI) by 38% from period 1 to 2. Mann-Whitney-Test of IR (BSI) per patient comparing period 1 and 2 gave a 2-tailed exact significance of p=0.098, hence supporting a tendency for reduced BSI in period 2. Table 3 (Tab. 3) shows the infection rates per period.

In period 1, 1 BSI (4%) was categorised as CVAD-related infection, 6 (21%) were categorised as CVAD-associated infection and 21 (75%) as secondary BSI. In period 2, 1 BSI (7%) were categorised as CVAD-related infection, 2 (13%) as CVAD-associated infection, and 13 (80%) as secondary BSI.

In Table 4 (Tab. 4) and Table 5 (Tab. 5) all pathogens detected in blood cultures are listed in detail. The corresponding proportions in period 1 were 57% (17/30) for Gram-positive and 43% (13/30) for Gram-negative pathogens, respectively. One BSI was caused by an ESBL-producing Gram-negative pathogen and one by *Candida parapsilosis*. Polymicrobial bacteraemia accounted for 11%. In period 2, 56% (10/18) of all pathogens detected in blood cultures were Gram-positive and 44% (8/18) were Gram-negative; 3 BSI (20%) were polymicrobial in origin. No methicillin-resistant *S. aureus *(MRSA) and no vancomycin-resistant *E. faecium* (VRE) were detected in blood cultures during the 42 months of surveillance at our paediatric treatment centre.

In terms of clinical severity, 17 of 28 (60%) BSIs in period 1 were graded as bacteraemia, 10 (36%) as sepsis and 1 (4%) as candidaemia. In period 2 11 of 15 (73%) were graded as bacteraemia, and 4 of 15 (27%) as sepsis.

In period 1 two BSI (7%) led to early removal of the device: 1 CVAD-related BSI (*E. cloacae*) as well as 1 secondary BSI event in which the CVAD was suspected, but not confirmed as the primary source of *P. aeruginosa *bacteraemia. During period 2, the CVAD was prematurely removed in one Broviac related BSI (6.7%) because of an infection caused by *E. faecium*. In both surveillance periods, none of the CVADs had to be removed prematurely due to a BSI caused by CoNS and persistent infection.

In all patients with detection of CoNS in 2 blood culture bottles drawn from a Broviac CVAD, the ethanol lock technique described previously [38], [39] was used as adjuvant local treatment in addition to systemic antibiotics (e.g. teicoplanin, administered through the CVAD) [40].

The median duration of inpatient treatment related to the infection was 8.5 days (IQR, 4–13 days; range, 3–28 days) in period 1 and 8.0 days (IQR, 6–6 days; range, 6–29 days) in period 2. During period 1, the BSI eventually contributed to death in 2 patients, one of them died due to septic shock with multiorgan-failure caused by an ESBL-producing *Enterobacter cloacae* infection. No BSI-related mortality was observed in period 2.

The retrospective analysis of alcoholic hand disinfectant (HD) consumption revealed an increase from 19 HDs per inpatient day (for one HD 3 ml of hand disinfectant were calculated) in 2010 to 28 and 38 HDs per inpatient day in 2012 and in 2013, respectively.

Using a very conservative approach (excluding costs of intensive care and costs of surgical interventions) a recent study from Germany calculated additional median costs of € 4,400 (IQR; € 3,145–5,920) per BSI event in paediatric oncology patients [41]. According to this model, the additional expenses due to the management of 28 BSIs in period 1 were estimated as € 123,200 (IQR; € 88,060–165,760). In period 2, the estimated additional cost for 15 BSI was € 66,000 (IQR; € 47,175–88,880).

During the 21 month of surveillance in period 2, the cumulative acquisition cost for the BD PosiFlush™ syringes in our in- and outpatient paediatric cancer facilities was € 27,422. In contrast, the acquisition cost of manually prepared sterile NaCl 0.9% flushing solutions would have been € 20,803, not counting the hands-on time of the healthcare professionals needed for the manual preparation. Thus, the absolute difference in material acquisition cost, related to the use of the BD PosiFlush™ syringes was € 6,619 in 21 months or € 3,782 per year. This investment per year is lower than the additional treatment cost of a single BSI [41]. In addition, the frequency of routine infusion system changes was reduced due to the new schedule (every 96 vs. every 48 hours). We did not systematically evaluate the consequences of this new schedule in terms of acquisition cost [16]. One complete i.v. system costs € 11 and the hands-on time required for one i.v. system change has been calculated previously with at least 25 minutes [16]. 

Taking material costs and nursing hours into consideration, relatively small changes in the number of i.v. system changes eventually result in significant cost savings. Last but not least, the frequency of routine flushing of the Broviac CVAD was reduced from 2 times a week to once weekly, which again reduced nursing hours and material consumption.

## Discussion

This study is the first report about the clinical evaluation of a modified preventive Broviac maintenance care protocol in a German paediatric oncology centre. The adjusted care protocol aims at the reduction of bloodstream infections in this high-risk population. Our study demonstrates a clinically relevant reduction in BSI incidence, incidence density and incidence rates. Especially the 75% reduction in CoNS BSI after introduction of our bundle together with the reduction of IR of all BSI per patient is strongly suggestive for its effectiveness in preventing infections associated with the maintenance care of CVADs. Although the combination of different procedures in a complex ‘bundle’ does not allow for the exact calculation of the impact related to single components [21], [22], [23], [42], reasonably chosen care protocols can be powerful drivers for the implementation of evidence-based care. Eventually this should result in an improvement of patients’ safety.

One limitation of our study may be that all bloodcultures were drawn exclusively from the long-term central venous catheter (Broviac) and not additionally from a peripheral vein. 

The comparative investigation of simultaneously sampled central and peripheral blood cultures in terms of differential time to positivity [43] adds to the early identification of the CVAD as the probable source of bacteraemia [44]. Without simultaneous peripheral venous cultures 14%–17% of all positive blood cultures remain undetected [45], [46]. In clinical practice, routine use of this technique is hampered by specific circumstances. Most notably patients and their parents are reluctant to tolerate additional pain and anxiety related to peripheral venous blood culture drawing in children with an easily accessible CVAD. This limits compliance with any written hospital-wide policy, recommending the collection of additional peripheral blood cultures. However this is consistent with clinical practice reports where peripheral venous blood cultures were sampled in only 58% of all cases [47]. Furthermore, the practical impact of this procedure on the choice and duration of antibiotic treatment is negligible in most cases [44], [48]. Hence supportive care recommendations published on behalf of the German Society for Paediatric Oncology and Haematology (GPOH) and the German Society for Paediatric Infectious Diseases (DGPI) do not recommend the additional collection of peripheral venous blood cultures from febrile paediatric cancer patients with a long term central venous catheter (CVAD) [18], [44], [49]. 

During the course of our investigation, four studies from the United States and one study from Spain have investigated the effect of different ‘bundles’ of CVAD maintenance care on BSI rates in paediatric cancer patients [21], [22], [23], [50], [51]. Although all studies (including ours) demonstrated a reduction in CVAD-associated infection rates following the implementation of a preventive maintenance care protocol, the difference between the 2 compared time periods was not significant in all studies [21]. This illustrates the restriction of single centre studies in paediatric cancer units with low CVAD-associated infection rates. A power calculation with our current results considering the incidence density of all BSIs revealed that we would have had to include more than 400 consecutive patients to reach a power of 80% (two-sided chi square test with a p<.05 significance level). Multicentre studies dealing with issues of supportive care are difficult to design, mainly because of the missing conformity in CVAD maintenance standards between centres [19], [52].

The next step may be the use of an antimicrobial locking solution [53], [54], [55] the invention of a Luer-lock split septum needleless connector [56] or of antimicrobial venous access caps [57]. 

On the other hand, one may argue that future studies should focus on the improvement of adherence to preventive maintenance care protocols instead of inventing further new technologies.

## Conclusions

The reduction of CVAD-associated BSIs in this study was clinically relevant but – probably due to insufficient power in a single centre observation – the difference did not reach statistical significance. Thus there is a need for multicentre studies investigating protocols to improve maintenance care of CVADs and reduce CVAD-associated bloodstream infections. This aim is of outstanding clinical relevance to increase our patients’ safety.

## Notes

### Competing interests

The study was designed, undertaken, and analyzed by the authors. BD (Becton, Dickinson and Company, Heidelberg, Germany) sponsored the data sampling of this clinical investigation. The commercial sponsor BD (Becton, Dickinson and Company, Heidelberg, Germany) had no role in study design, data collection, data analysis, data interpretation, or writing of the report. All authors had full access to all data in the study and had final responsibility for the decision to submit for publication. AS has been invited to and participated in advisory boards on vascular catheter care organized by BD. Other authors: no conflict of interest to declare.

### Acknowledgement

We thankfully acknowledge the clinical work of Thomas Krenn, Daniela Lothschütz and Sabine Heine, paediatric oncology, haematology and coagulation disorder consultants in our unit and the tremendous every day work of our complete paediatric oncology nursing team.

## Figures and Tables

**Table 1:**
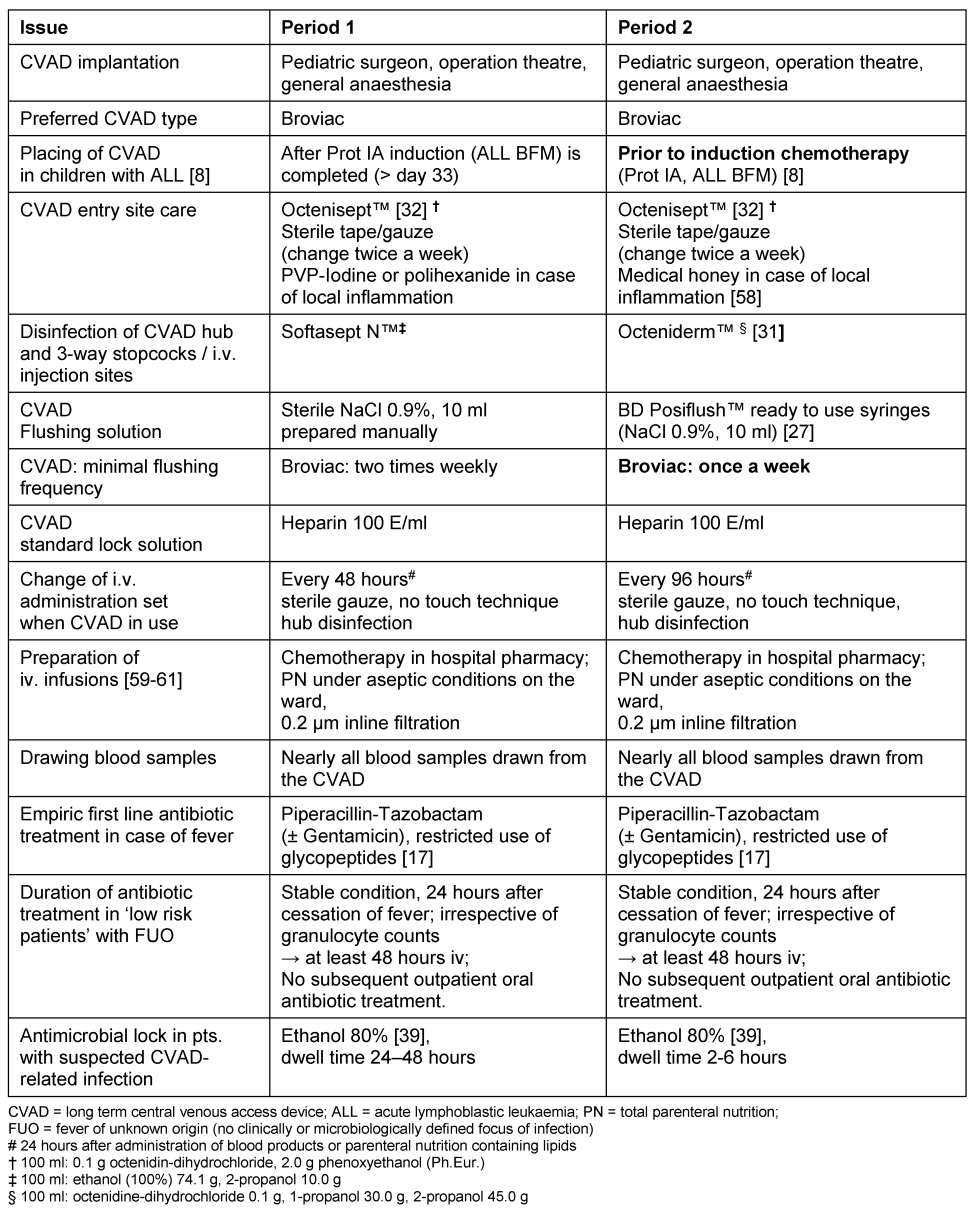
Comparison of CVAD care and maintenance practices to prevent bacteraemia

**Table 2:**
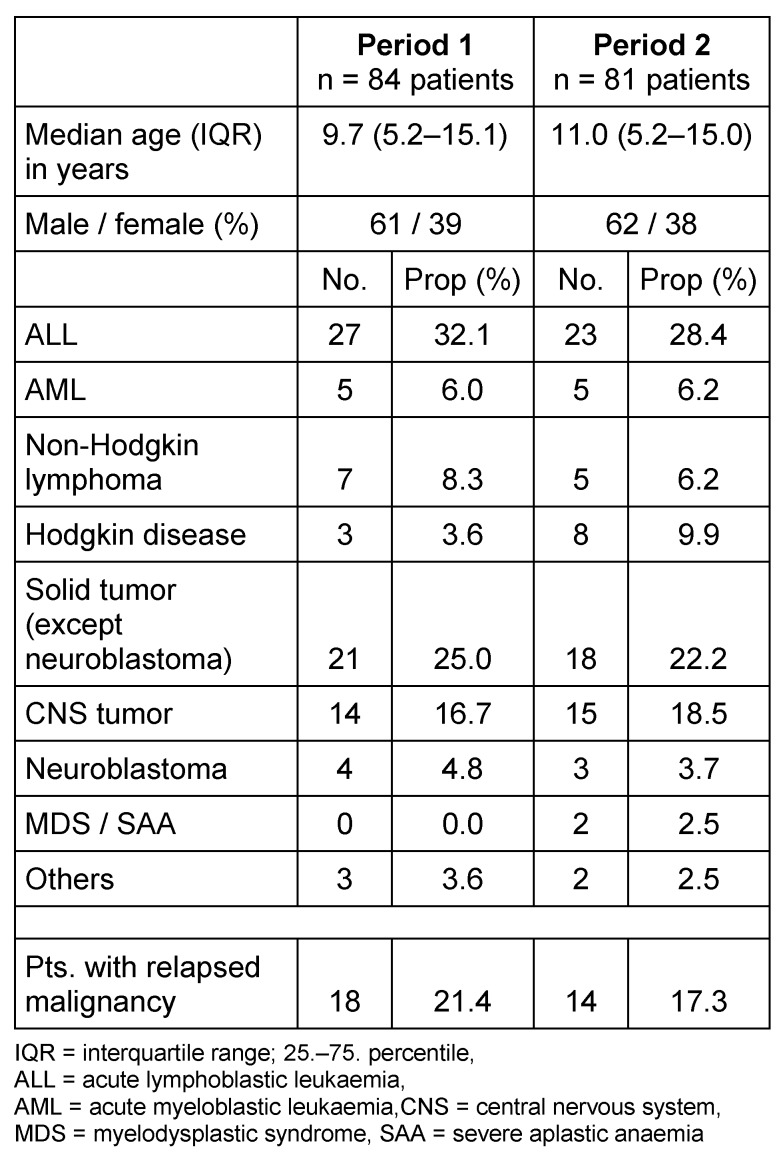
Basic patient characteristics in 2 consecutive surveillance periods. Testing the disease incidences (Fisher’s Exact) in the 2 groups showed no significant differences in all cases.

**Table 3:**
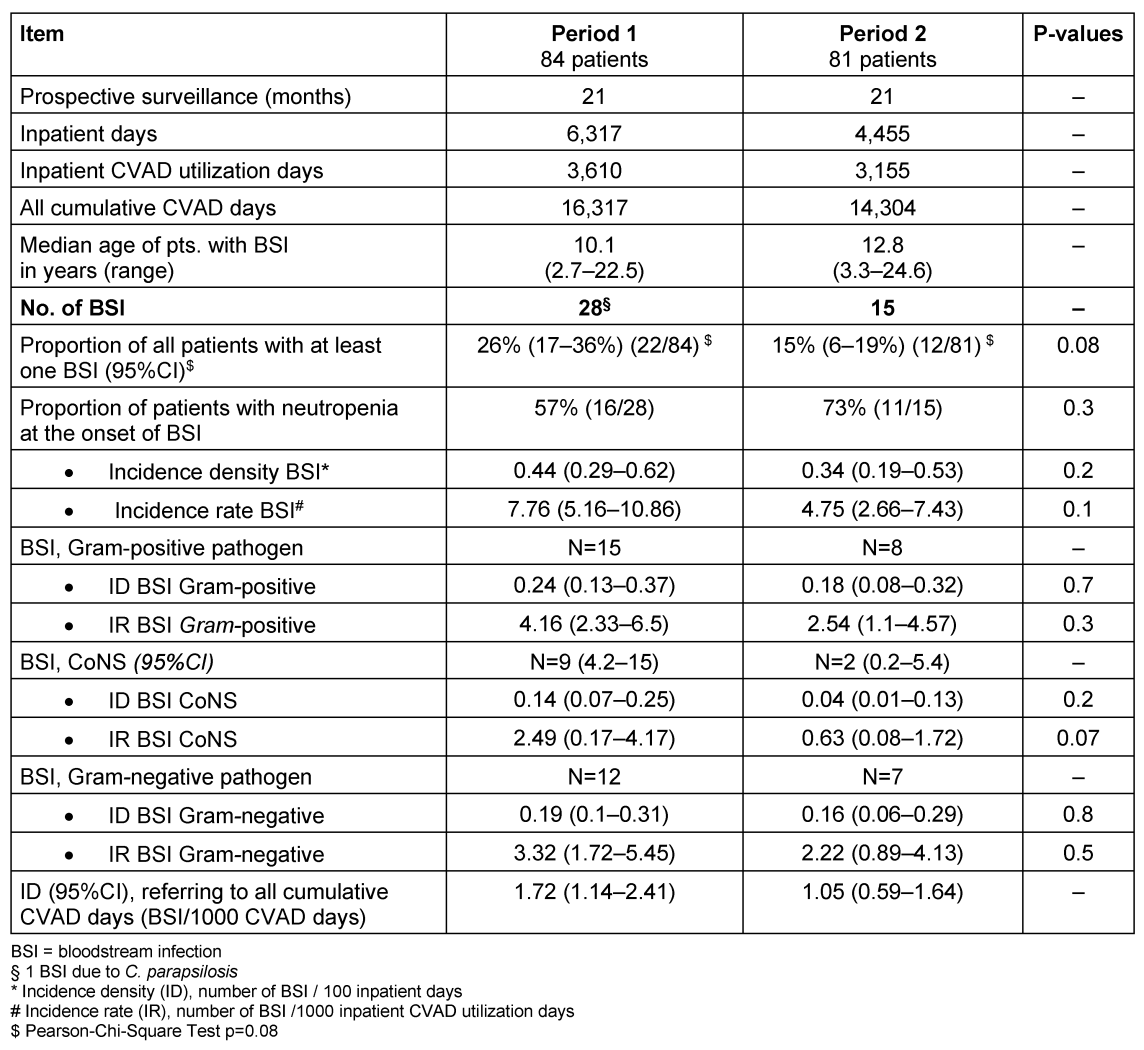
Bloodstream infections (BSI) and corresponding infection rates

**Table 4:**
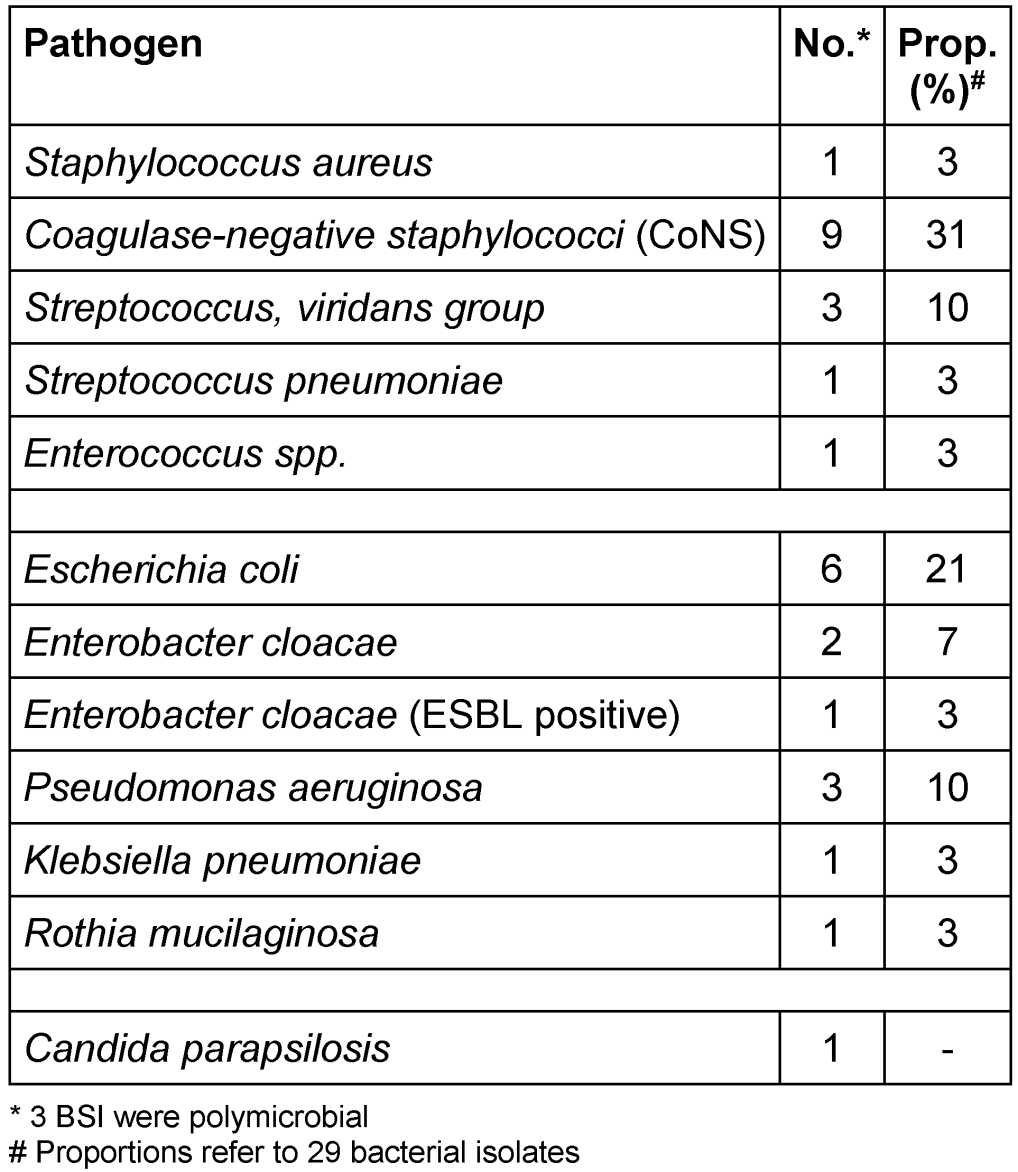
Period 1: Pathogens detected in 28 BSI (22 patients)

**Table 5:**
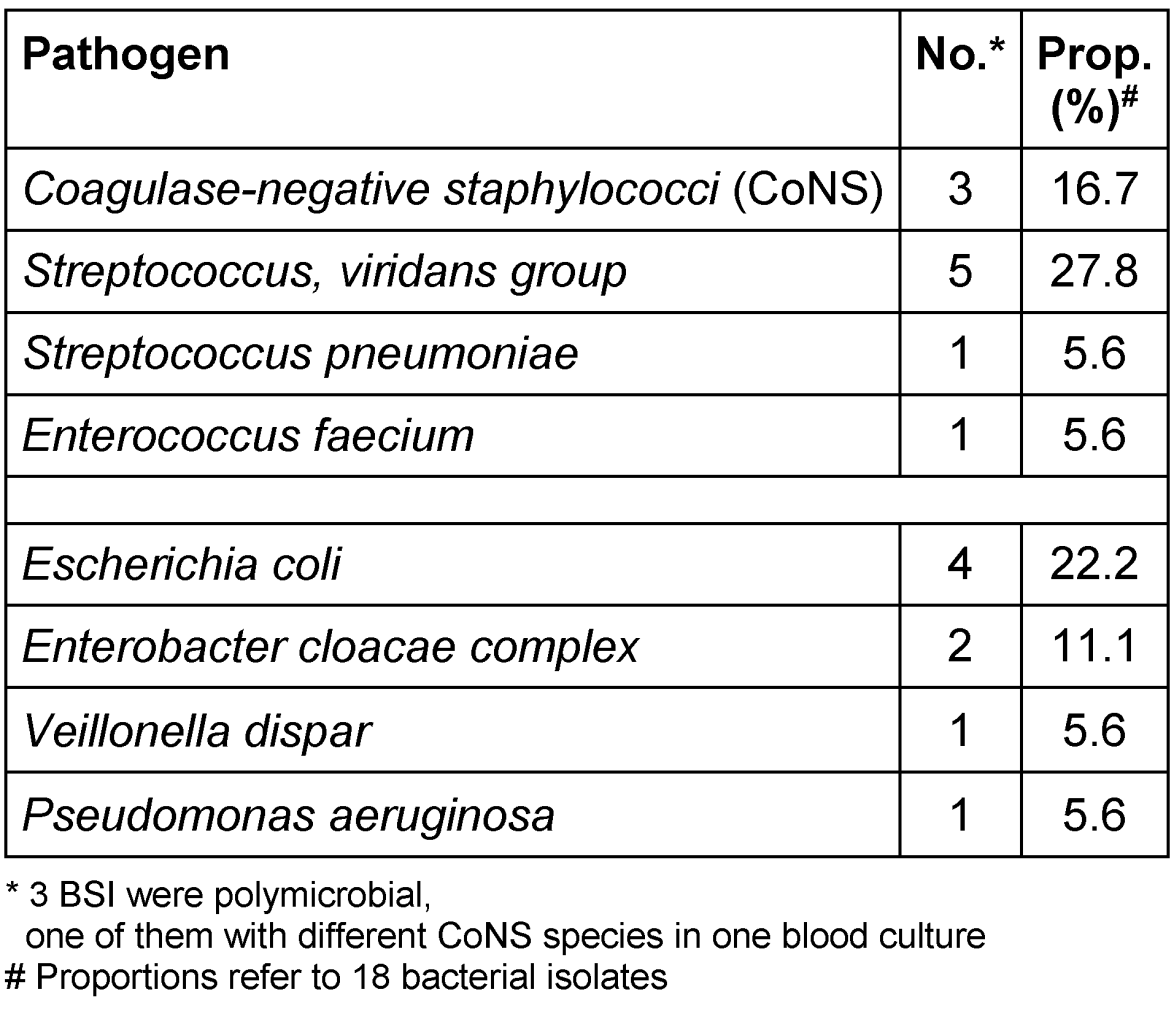
Period 2: Pathogens detected in 15 BSI (12 patients)
